# Combination of antibodies directed against different ErbB3 surface epitopes prevents the establishment of resistance to BRAF/MEK inhibitors in melanoma

**DOI:** 10.18632/oncotarget.4485

**Published:** 2015-07-07

**Authors:** Luigi Fattore, Debora Malpicci, Emanuele Marra, Francesca Belleudi, Alessia Noto, Claudia De Vitis, Maria Elena Pisanu, Pierpaolo Coluccia, Rosa Camerlingo, Giuseppe Roscilli, Antoni Ribas, Arianna Di Napoli, Maria Rosaria Torrisi, Luigi Aurisicchio, Paolo Antonio Ascierto, Rita Mancini, Gennaro Ciliberto

**Affiliations:** ^1^ Istituto Nazionale per lo Studio e la Cura dei Tumori “Fondazione G. Pascale”, Naples, Italy; ^2^ Dipartimento di Chirurgia “P. Valdoni”, Sapienza Università di Roma, Rome, Italy; ^3^ Dipartimento di Medicina Sperimentale e Clinica, Università degli Studi di Catanzaro “Magna Graecia”, Catanzaro, Italy; ^4^ Takis S.r.l., Rome, Italy; ^5^ Dipartimento di Medicina Clinica e Molecolare, Sapienza Università di Roma, Rome, Italy; ^6^ Istituto Pasteur Fondazione Cenci Bolognetti, Dipartimento di Medicina Clinica e Molecolare, Sapienza Università di Roma, Rome, Italy; ^7^ Department of Medicine, Division of Hematology/Oncology, University of California Los Angeles (UCLA), Los Angeles, CA, USA; ^8^ Azienda Ospedaliera S. Andrea, Rome, Italy

**Keywords:** melanoma, BRAF/MEK inhibitors, ErbB3 activation, Anti-ErbB3 antibodies, *in vivo* regrowth impairment

## Abstract

Patients with metastatic melanoma bearing V600 mutations in BRAF oncogene clinically benefit from the treatment with BRAF inhibitors alone or in combination with MEK inhibitors. However, a limitation to such treatment is the occurrence of resistance. Tackling the adaptive changes helping cells survive from drug treatment may offer new therapeutic opportunities. Very recently the ErbB3 receptor has been shown to act as a central node promoting survival of BRAF mutated melanoma. In this paper we first demonstrate that ErbB3/AKT hyperphosphorylation occurs in BRAF mutated melanoma cell lines following exposure to BRAF and/or MEK inhibitors. This strongly correlates with increased transcriptional activation of its ligand neuregulin. Anti-ErbB3 antibodies impair the establishment of *de novo* cell resistance to BRAF inhibition *in vitro*. In order to more potently ablate ErbB3 activity we used a combination of two anti-ErbB3 antibodies directed against distinct epitopes of its extracellular domain. These two antibodies in combo with BRAF/MEK inhibitors potently inhibit *in vitro* cell growth and tumor regrowth after drug withdrawal in an *in vivo* xenograft model. Importantly, residual tumor masses from mice treated by the antibodies and BRAF/ERK inhibitors combo are characterized almost exclusively by large necrotic areas with limited residual areas of tumor growth. Taken together, our findings support the concept that triple therapy directed against BRAF/MEK/ErbB3 may be able to provide durable control of BRAF mutated metastatic melanoma.

## INTRODUCTION

Malignant melanoma is the most aggressive form of skin cancer. Its incidence has dramatically increased wordwide over the past decades, thus becoming a major medical problem [1]. Although historical survival rates for patients with metastatic melanoma have been low until recently [2, 3], clinical management of this disease has significantly improved over the last 3–4 years thanks to the introduction of two classes of drugs: a) immunological checkpoint inhibitors such as monoclonal antibodies against CTLA-4 and PD-1/PD-L1 [4]; b) small molecule kinase inhibitors of the RAS/RAF/MAPK pathway for the approximately 50% of patients bearing mutations of the BRAF oncogene [5]. BRAF mutations usually affect the Valine 600 codon changing this aminoacid into glutamic acid (V600E) in the majority of cases, but also, less frequently, into other aminoacids (V600D, V600R) [6]. These mutations cause the constitutive activation of the BRAF kinase, which aberrantly induces MAPK/ERK kinases [6]. Disease prognosis for melanoma patients bearing BRAF V600 mutations has drastically improved in relation to the introduction of BRAF inhibitors (BRAFi) two of which, vemurafenib and dabrafenib, have already been approved by FDA [7, 8]. BRAF inhibitors are active only in tumors where V600 BRAF mutations result in constitutively active monomers, whereas the same inhibitors induce paradoxical tumor promoting effects in RAS mutated melanomas because of their ability to promote allosteric activation through homo- or hetero-dimerization of wild type B RAF isoforms [9, 10]. Although BRAFi induce unprecedented objective responses in approximately 45 to 50% of treated patients, virtually all responders undergo disease progression within 5 to 6 months after initiation of treatment as a consequence of the development of *de novo* drug resistance [11, 12]. The mechanisms at the basis of acquired resistance have been at the center of intensive investigations. These have led to discover in the majority of cases a plethora of mutations which cause reactivation of the RAS/RAF/MAPK pathway, including NRAS or KRAS mutations, mutant BRAF amplifications, alternative BRAF splicing, MAP2K1 activating mutations and CDKN2A losses [13–16].

The evidence that resistance to BRAFi is caused by reactivation of the MAPK pathway has led to the development of novel strategies directed to simultaneously inhibit BRAF and the downstream MEK kinase in the attempt to reduce the emergence of resistance. Indeed, MEK inhibitors increase objective response rates, progression free survival and, more recently, overall survival when delivered in combination with a BRAF inhibitor as compared to BRAF inhibitor monotherapy [17–20]. Thus combination therapy is expected to become soon the standard of care for this subset of patients. However also this approach is unable to completely eradicate disease and disease progression occurs after an average of approximately 10 months [21]. Alternative mechanisms of resistance are related to the activation of signaling pathways redundant to MAPK, for example overexpression of RTKs, such as PDGFR or IGF1R, which promote activation of the PI3K-AKT axis [22–24]. These mechanisms have been observed both in melanoma cell cultures exposed *in vitro* to continuous selection with BRAF inhibitors, and in post-relapse human melanoma tumor samples [14].

An alternative approach to the study of drug resistance is the analysis of early adaptive changes taking place in cells shortly after drug exposure. We believe that a better knowledge of these early events may help develop new strategies aiming at circumventing the establishment of drug resistance. Using this approach our laboratory as well as others have recently shown that the ErbB3 receptor is involved in the activation of an early feedback survival loop soon after drug exposure which leads to increased phosphorylation of the prosurvival AKT kinase [25–27]. A distinguishing feature of our observations was that, upon exposure to BRAF or MEK inhibitors, ErbB3 does not undergo transcriptional activation but instead a selective increase of its phosphorylation consequent to enhanced autocrine production of its ligand neuregulin-1 (NRG1) [27]. Also, we were the first to show that activation of the ErbB3/AKT axis can be inhibited by co-treatment with anti-ErbB3 mAbs [27] and that these antibodies synergize with BRAF and MEK inhibitors in short term *in vitro* clonogenic assays [27]. We had previosuly generated a group of monoclonal antibodies directed against the extracellular domain of human ErbB3 [28] and shown that two of these antibodies, named A3 and A4, were able to inhibit receptor phosphorylation and melanoma cell growth mainly through the induction of receptor internalization and degradation [29, 30].

In the present work we have addressed two important questions. In first instance we have decided to assess whether the ErbB3 autocrine survival loop induced by treatment with BRAF and/or MEK inhibitors is a general phenomenon using a larger panel of melanoma cell lines and whether *in vitro* treatment with anti-ErbB3 antibodies is capable of suppressing the establishment of resistance to BRAFi. The second question we have addressed is whether a combinatorial approach with two different antibodies directed against distinct ErbB3 surface epitopes can better inhibit receptor function in BRAF mutated melanomas. Previous studies focusing on two other receptors of the same family, namely EGFR and ErbB2, have shown that a stronger inhibition of their protumorigenic signaling activity was achieved with a combination of two antibodies against different surface epitopes. This approach was more powerful due to a pronounced ability to induce receptor downmodulation and redirection into the lysosomal degradative pathway [31]. Indeed here we show both *in vitro* and in an *in vivo* xenograft model that combined treatment with A3 and A4 antibodies is able to more potently induce receptor degradation and as a result to more potently sinergize with the BRAF/MEK inhibitors in tumor growth impairment and induction of apoptosis.

## RESULTS

### ErbB3 receptor is rapidly phosphorylated in a broad panel of BRAF-mutated melanoma cell lines upon exposure to a BRAF or to a MEK inhibitor

We and others have recently shown that the ErbB3 receptor is involved in the activation of an early feedback survival loop upon cell exposure to BRAF and/or MEK inhibitors [25–27]. Two different mechanisms have been postulated. In first instance Abel et al [26] suggested that BRAF mutated melanoma cell lines, when exposed to a BRAF inhibitor, undergo rapid increase of ErbB3 mRNA transcription/translation (1.5- to 3-fold) which makes cells more sensitive to stimulation by exogenously added ligand Neuregulin-1 (HRG or NRG1). In contrast to this finding we observed in three cell lines with different BRAF V600 mutations, namely MST-L, LOX-IMVI and WM266, that cell exposure to either vemurafenib as BRAF inhibitor or trametinib as MEK inhibitor, does not lead to increase ErbB3 protein expression but instead causes increased spontaneous phosphorylation of ErbB3 (several-fold) [27]. In one cell line we showed that this is consequent to increased endogenous production of NRG1 and activation of an autocrine loop [27].

In order to confirm and expand our previous observations we conducted an extensive analysis of a larger panel of 11 BRAF-mutated melanoma cell lines indicated in Table 1. In all cell lines we initially assessed the level of expression of all ErbB receptors and found that in all of them, ErbB3 was constantly expressed at high levels with the exception of M263 cells. In contrast all other ErbB receptors were expressed at variable levels with ErbB1/EGFR and ErbB4 showing low or undetectable levels in several cells.

**Table 1:** ErbB3 receptor is phosphorylated in a panel of BRAF-mutated melanoma cell lines upon exposure to a vemurafenib and/or trametinib

Cell line	ErbB1	ErbB2	ErbB3	ErbB4	Activation of pErbB3 by BRAFi and vemurafenib IC50	Activation of pErbB3 by MEKi and trametinib IC50
M14 (V600E)	0	70%	71%	47%	+ / 0.35 μM	+ / 0.21 μM
LOXIMVI (V600E)	2%	42%	71%	78%	+ / 0.15 μM	+ / 0.11 μM
A375 (V600E)	70%	1%	1%	49%	+ / n.d.	+ / n.d.
SK Mel5 (V600E)	29%	38, 1%	42, 1%	93, 7%	+ / n.d.	− / n.d.
M262 (V600E)	2%	15%	47%	11%	+ / 0.2 μM	+ / n.d.
M263 (V600E)	0	1%	10%	0	− / n.d.	− / n.d.
M229 (V600E)	0	28%	97%	1%	+ / 0.3 μM	+ / n.d.
M397 (V600E)	0	7%	79%	0	+ / n.d.	+ / n.d.
WM266 (V600D)	33%	66%	68%	60%	+ / 0.16 μM	+ / 0.1 μM
WM115 (V600D)	9%	34%	51%	14%	+ / n.d.	+ / n.d.
MST-L (V600R)	1%	9%	39%	48%	+ / 0.26 μM	+ / 0.17

Flow cytometry analysis of ErbBs membrane expression in a panel of 11 melanoma cell lines. The percentage of positive cells was determined by staining with the indicated primary antibodies and with the isotype-matched andibodies as negative control. ErbB3 is constantly expressed at high levels with the exception of M263 cells. Cell extracts from these cell lines exposed to vemurafenib and/or trametinib were prepared and subjected to western blotting. The results summarized in this Table show that ErbB3 is activated in 10 out of 11 cell lines evaluated upon cells exposure to drugs. The IC50 values relative to vemurafenib or trametinib was assessed through *in vitro* proliferation assays as described in the materials and methods section.

**Figure 1:**
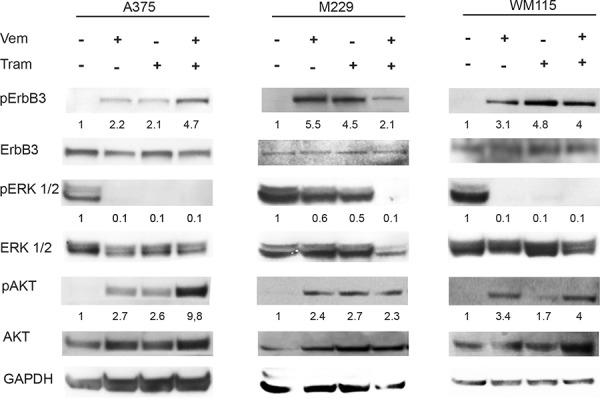
Vemurafenib and/or Trametinib treatments induce selective ErbB3 phosporylation and AKT activation in melanoma cells A375, M229 and WM115 cells were serum starved for 24 h, treated or not with vemurafenib (0.3 μM), with trametinib (0.15 μM) or with their combination for 24 h. Western blot analysis performed using the indicated antibodies shows that both vemurafenib and trametinib induce a strong phosphorylation of ErbB3 (Y1289) and AKT (S473). For densitometric analysis results are expressed as mean values from three independent experiments.

Cell extracts of the 8 new melanoma cell lines M14, A375, SK-Mel5, M262, M263, M229, M397 and WM115 exposed to vemurafenib and/or trametinib were prepared and subjected to western blotting. The results, summarized in Table 1, show that in the majority of cell lines ErbB3 undergoes a strong upregulation of its phosphorylation in the absence of external addition of NRG1 upon exposure to vemurafenib or trametinib or both drugs. Detailed results of three positive cell lines (A375, M229 and WM115) are shown in Figure 1. Notably, feedback activation of pErbB3 is accompanied by strongly increased phosphorylation of AKT (Figure 1). In summary, while we observed upregulation of ErbB3 phosphorylation in 10 out of 11 cell lines, we did not observe any significant increase in ErbB3 protein levels in agreement with our previous report [27]. In order to further investigate this aspect we carried out qPCR analysis of ErbB3 and NRG1 mRNA levels in the “positive” cell lines after exposure to vemurafenib. The results (Supplementary Table S1) show that ErbB3 mRNA levels remain substantially unchanged, whereas NRG1 mRNA levels constantly increase from a minimum of 1.7-fold to a maximum of 7-fold in all cells analyzed. We also determined whether vemurafenib plus trametinib is able to induce NRG1 transcription in the three cell lines of Figure 1. The results (Supplementary Figure S1), show that also the combination treatment is able to induce autocrine NRG1 production albeit at variable levels in the various cell lines. Incidentally, A375 cells present the highest amplitude of NRG1 induction and also show the strongest degree of AKT phosphorylation.

It has been recently reported that EPHA2 is involved in the establishment of resistance to vemurafenib via upregulation of its expression and increased levels of S897 phosphorylation [32, 33]. EPHA2 S897 hyperphosphorylation in resistant cells is mediated by AKT [32]. Hence, we decided to assess whether also in sensitive cell lines AKT S473 hyperphosphorylation following short term treatment with BRAFi and/or MEKi correlated with increased levels of S897 EPHA2. Surprisingly, in sensitive cells (Supplementary Figure S2) EPHA2 S897 is shut off at the same time S473 AKT is upregulated. This finding suggests that EPHA2 is not involved in the early adaptive survival loop downstream to ErbB3.

In order to assess whether anti-ErbB3 monoclonal antibodies are capable of inhibiting vemurafenib and trametinib upregulation of pErbB3 and pAKT, cells were simultaneously exposed to treatment combinations with anti-ErbB3 antibody A3 and cell extracts subjected to Western blotting. The results (Supplementary Figure S3) show, as expected, that A3 is able to partially but reproducibly decrease vemurafenib/trametinib-induced activation of the pErbB3/pAKT pathway.

### ErbB3 mAbs inhibit the establishment of resistance and restore drug sensitivity to vemurafenib resistant melanoma cells

A critical issue in the management of BRAF mutated melanoma is the development of resistance to BRAF inhibitors [33]. In most cases resistance has been linked to the reactivation of the MAPK/ERK pathway as consequence to secondary mutations or altered protein expression and it has been shown that these cells can still be growth-impaired by MEK kinase inhibitors both *in vitro* and in the clinic [17, 35].

**Figure 2:**
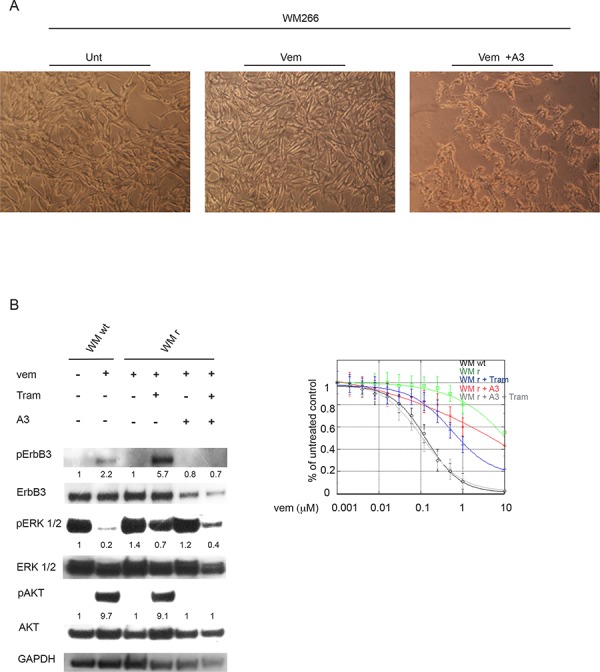
Anti-ErbB3 mAbs restore drug sensitivity to vemurafenib in resistant melanoma cells WM266 human melanoma cells were treated for about two months with increasing drug concentrations every two weeks (from 50 nM to 10 μM) in order to generate vemurafenib resistant cells. **A.** Magnifications of cells show that the combination of vemurafenib (at the initial concentration of 50 nM) with the anti-ErbB3 antibody A3 (at the concentration of 10 μg/ml) decreases cell viability compared to untreated (Unt) or vemurafenib-treated (Vem) after two week of treatment. The pictures shown are representative of three indipendent experiments. **B.** WM266 melanoma cells resistant to vemurafenib (WM r) were serum starved and treated with vemurafenib (0.3 μM), with trametinib (0.15 μM) or with their combination in presence or not of anti-ErbB3 mAb A3 (20 μg/ml) for 24 h. Western blot analysis shows that A3 mAb abrogate ErbB3 phosphotylation (Y1289) as well as the strong increase of pAKT (S473) induced by trametinib in vemurafenib resistant WM266 cells (WM r). WM266 wild type cells (WM wt) were used as positive control. For densitometric analysis results are expressed as mean values from three independent experiments. **C.** WM r cells were grown in the presence of different doses of vemurafenib and combined or not with trametinib (0.15 μM), A3 mAb (20 μg/ml) or their combination for 10 day. Cells were stained with crystal violet and then dissolved in a Methanol/SDS solution and the adsorbance (595 nm) was read using a microplate ELISA reader. Quantitative analysis for curve fitting and for IC50 evaluation, performed by KaleidaGraph software, shows that either trametinib (at a greater extent) or A3 (at a lower extent) are able to partially restore cell sensitivity to vemurafenib, but only the co-treatment of the resistant clones with both molecules is able to fully restore cells sensitivity to vemurafenib. IC50 Vem WM wt = 0.16 μM; IC50 Vem WM r = 7 μM; IC50 Vem WM r + Tram = 0.77 μM; IC50 Vem WM r + A3 = 3 μM; IC50 Vem WM r + Tram + A3 = 0.1 μM. *p*-values were calculated using Student's *t* test and significance level has been defined as *p* < 0.05. For IC50 Vem WM r + Tram + A3 *p* < 0.001 vs IC50 Vem WM r cells. For IC50 Vem WM r + Tram *p* < 0.05 vs IC50 Vem WM r cells. For IC50 Vem WM r + A3 NS vs IC50 Vem WM r. WM wt were used as control.

We sought initially to generate cells resistant either to vemurafenib or to the combination of vemurafenib plus one of our anti-ErbB3 mAbs by exposing WM266 cells to growing concentrations of vemurafenib. As shown in Figure 2A, while we were able to select cells resistant to vemurafenib, long term co-treatment with combination of vemurafenib (at the initial concentration of 50 nM) and of the anti-ErbB3 antibody A3 (at the concentration of 10 μg/ml) always resulted in a strong decrease in cell viability and in visible stress-induced changes in cell morphology (see Figure 2 rightmost panel) which did not allow to continue the selection process after 1–2 weeks of culture. Of notice, treatment of cells with A3 alone did not induce any significant effect on cell viability (data not shown). This experiment was repeated at least three times also with a different anti ErbB3 antibody with similar results. Hence we can conclude that blocking ErbB3 together with BRAF strongly affects the development of *de novo* resistance.

On the other hand, using growing concentrations of vemurafenib up to 10 μM we were able to obtain a population of cells resistant to this drug. We used these cells to analyze ErbB3 expression and activation, and whether anti-ErbB3 antibodies could still be able to synegize with trametinib.

Total protein extracts of WM266 resistant cells exposed to A3, vemurafenib and/or trametinib were subjected to Western Blot analysis. The results (Figure 2B) show that these cells maintain the ability to undergo pErbB3 activation upon short term exposure to trametinib and that this is abolished by co-treatment with the A3 mAb. Importantly, A3 treatment in combination vemurafenib and trametinib in resistant cells is able to further reduce pERK activation.

In addition, *in vitro* colony formation assays were carried out on WM266 resistant clones in the presence of growing concentrations of vemurafenib alone or in combination with A3 and/or trametinib. Our results clearly show that, while treatment of vemurafenib resistant cells with either trametinib (at a greater extent) or A3 (at a lower extent) is able to partially restore cell sensitivity to vemurafenib, only the co-treatment of the resistant clones with both trametinib and A3 is able to fully restore cells sensitivity to vemurafenib (Figure 2C, right panel). The results described above led us to postulate that suppressing the RAS-RAF-MAPK pathway at multiple levels in combination with anti-ErbB3 mAbs is able to control and reduce cancer growth for a longer time by inhibiting the early and the long-term adaptive mechanisms centered around ErbB3.

### Combination of two anti-ErbB3 mAbs binding different surface epitopes strongly inhibits melanoma cell growth and receptor internalization and degradation

It has been previously shown that combining pairs of anti-ErbB mAbs recognizing distinct epitopes in the extracellular domain exerts a superior antitumor effect compared to the use of individual antibodies because of their ability to inhibit receptor recycling [31, 36]. We therefore assessed the effect of the two anti-ErbB3 antibodies A3 and A4. We have previously shown that both antibodies inhibit NRG1 binding, NRG1-induced pHER3 stimulation, ligand-induced signaling and ligand-induced melanoma cell proliferation through binding to different epitopes [29, 30], one of which (A3) has been mapped to the ErbB3 heterodimerization loop with other ErbBs.

**Figure 3:**
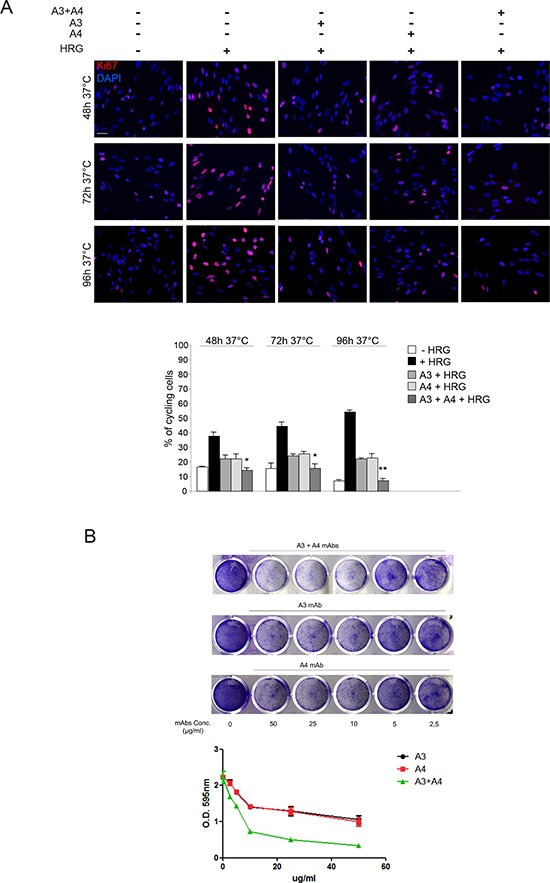
Combination of anti-ErbB3 mAbs is more effective in inhibiting melanoma cell growth compared to the single treatments **A.** MST-L cells were pre-treated with each mAb or their combination and then incubated with NRG1 (HRG) for 48, 72 and 96 h in the presence of the antibodies, fixed and stained with anti-Ki67 antibodies to identify cycling cells. Quantitative analysis of the percentage of cells presenting Ki67-positive nuclei was performed as reported in materials and methods and values are expressed as mean values ± standard errors (SE). Student's *t* test was performed and significance level has been defined as described in materials and methods. The increase of the proliferation rate upon 48, 72 and 96 h of NRG1 stimulation is inhibited either by mAbs combination and A3/A4 individually. Cell growth inhibition induced by mAbs combination is particularly pronounced after 96 h of treatment comapred to single mAbs. *p < 0.05 vs. the corresponding NRG1-treated cells, **p < 0.001 vs. the corresponding HRG-treated cells. Bar = 10 μm. **B.** MST-L cells were grown in the presence of different doses A3 and A4 alone or in combination for 10 days. Cells were then fixed and stained with crystal violet (upper part). Cells were then dissolved in a Methanol/SDS solution and the adsorbance (595 nm) was read using a microplate ELISA reader. Quantitative analysis for curve fitting and for IC50 evaluation, performed by GraphPad software, shows that A3 and A4 combination is able to inhibit melanoma cell growth better than the single treatments. EC50 A3 = 38 μg/mL; EC50 A4 = 35 μg/mL; EC50 A3 + A4 = 7 μg/mL. The Combination Index (CI) evaluation, performed by CalcuSyn software as reported in materials and methods, indicate that A3 and A4 are synersistic drugs; CI = 0.7.

For this purpose we used MST-L cells which are known to be growth stimulated upon exposure to exogenous NRG1. First of all we compared the proliferative response of MST-L melanoma cells pre-treated with each mAb alone or their combination and then incubated with NRG1 (HRG) for 48, 72 and 96 h in the presence of the mAbs. After incubation, cells were fixed and stained with anti-Ki67 antibodies to identify cycling cells. Quantitative analysis of the percentage of cells presenting Ki67-positive nuclei indicated that the increase of the proliferation rate, clearly evident upon 48, 72 and 96 h of NRG1 stimulation, was strongly and significantly inhibited by the mAbs combination compared to single treatment with either A3 or A4 mAbs individually. Melanoma cell growth inhibition induced by mAbs combination compared to single mAbs was particularly pronounced after 96 h of treatment (Figure 3A).

In order to confirm these data, we performed *in vitro* colony formation assays in the presence of growing concentrations of A3 and A4 alone or in combination for ten days. Remarkably A3 + A4 combination was able to better inhibit MST-L melanoma cells growth compared to the single treatments (Figure 3B).

**Figure 4:**
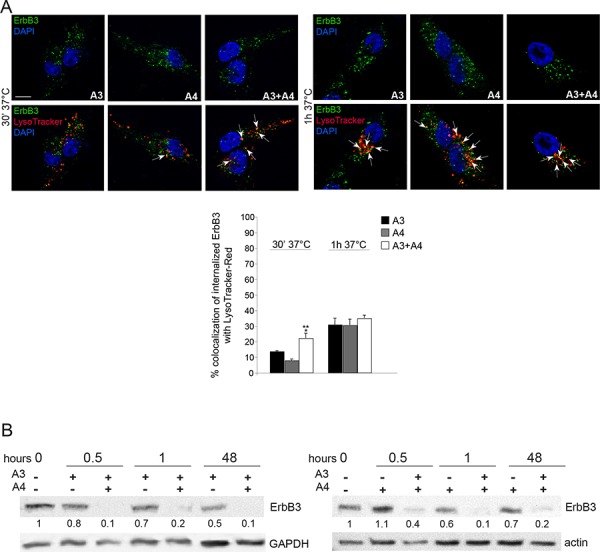
Combination of anti-ErbB3 mAbs is more effective in inducing receptor internalization and degradation **A.** MST-L cells were treated with A3, A4 or their combination for 30′ and 1 h at 37°C in the presence of LysoTracker-Red as reported in materials and methods. Quantitative immunoflorescence analysis of the percentage of colocalization of signals and 3D reconstruction was performed as described in materials and methods. Results are expressed as mean values ± SE (standard errors): the percentage of colocalization was calculated analyzing a minimum of 50 cells for each treatment randomly taken from three independent experiments. Student's *t*-test was performed and significance level has been defined as described in materials and methods. A colocalization of ErbB3-bound A3, A4 and their combination with the LysoTracker marker in intracellular, perinuclear dots corresponding to lysosomes is evident. mAbs combination strongly accelerate receptor targeting to the lysosomal compartment compared to single treatments. **p < 0.001 vs. the corresponding A3 or A4-treated cells; the slight increase of colocalization of combination compared to single treatments at 1 h is not significant. Bar = 10 μm. **B.** Western blot analysis using anti-ErbB3 polyclonal antibodies in MST-L cells treated with A3, A4 or their combination at 37°C for the different time points (0.5, 1, 48 h). A drastic decrease of the band corresponding to ErbB3 is evident after mAbs combination treatments compared to single mAbs. The equal loading was assessed with anti-actin antibody and densitometric analysis was performed as described in materials and methods.

Since we have previously demonstrated that A3 and A4 mAbs are able to potently induce ErbB3 internalization and degradation by inducing receptor relocalization to the endocytic lysosomal degradative pathway [29], we decided to investigate if mAbs combination might be more effective compared to single treatments. In order to clarify this aspect MST-L cells were treated with A3, A4 or with A3 + A4 for 1 h at 37°C in the presence of LysoTracker-Red to identify the lysosomal compartment. Quantitative immunofluorescence analysis demonstrated colocalization of ErbB3-bound A3, A4 and A3 + A4 with the LysoTracker marker in intracellular, perinuclear dots corresponding to lysosomes, according to our previous data [29]. Moreover, we clearly observed that the mAbs combination is able to strongly accelerate receptor relocalization to the lysosomal compartment compared to single mAbs, especially after short treatments (Figure 4A, arrows). In order to verify whether the accelerated ErbB3 redistribution to the endocytic degradative pathway induced by A3 and A4 combination would actually correspond to more efficient and rapid receptor degradation compared to single treatments, we performed Western Blot analysis. Results (Figure 4B) show a dramatic decrease of the ErbB3 signal already after 30 minutes and 1 h of treatment with the A3 + A4 combination compared to single treatments. It is of interest to notice that also after 48 h only the combined treatment with A3 + A4 is able to maintain persistent downregulation of ErbB3.

Finally, in order to identify the mechanism responsible for mAbs-induced ErbB3 internalization we treated MST-L cells with NRG1, A3 or A4 for different time points and subjected total protein extracts to immunoprecipitations with an antibody directed against the ErbB3 receptor. Western Blot analysis using anti-UBI antibodies showed that A3 and A4 induce strong ErbB3 receptor ubiquitination after 30 minutes of treatment compared to NRG1 stimulation (Supplementary Figure S4a). To further demonstrate the involvement of the ubiquitination process in mAbs-induced receptor internalization we exposed MST-L cells to the ubiquitination-inhibitor PYR-41 and then to A3, A4 or NRG1. To evaluate the amount of ErbB3 receptors that remained localized on the plasma membrane upon the treatments described above, internalization experiments were performed. The plasma membranes were visualized by incubating the cells with the lipophilic tracer Vybrant DiI at 4°C before fixation. Immunofluorescence analysis showed that, PYR-41 pre-treatment was able to block mAbs and ligand-induced receptor internalization (Supplementary Figure S4b). Moreover, PYR-41 pre-treatment inhibited mAbs-induced ErbB3 degradation and ubiquitination as showed by Western Blot and immunoprecipitation analysis in Supplementary Figure S4c and S4d.

### Anti-ErbB3 mAbs combination with vemurafenib and/or trametinib inhibits proliferation and induces apoptosis better than single antibody treatments

**Figure 5:**
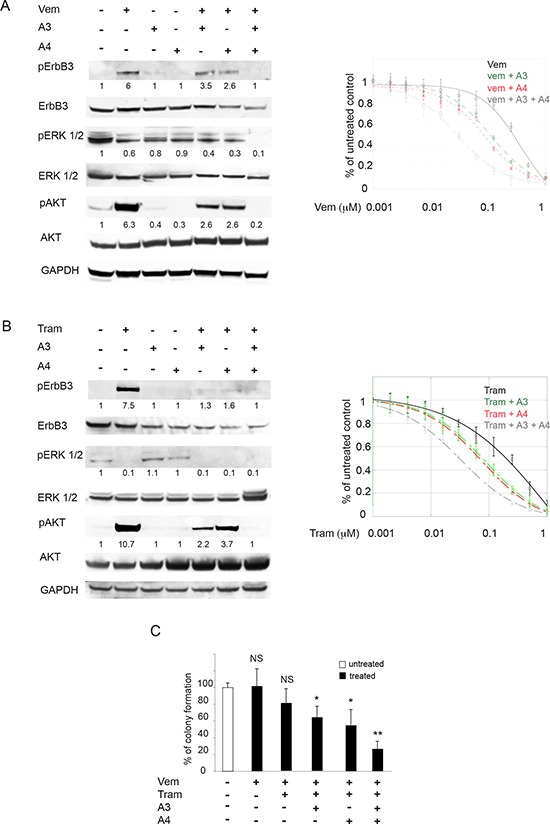
Anti-ErbB3 mAbs combination counteracts the increase of receptor phosphorylation and potentiate growth inhibition induced by vemurafenib and trametinib better than the single mAbs M14 melanoma cells serum starved and treated with vemurafenib (0.3 μM) **A. left part** or trametinib (0.15 μM) **B. left part**) for 24 h were incubated with 20 μg/ml of anti-ErbB3 mAbs A3, A4 or their combination. Western blot analysis shows that only mAbs combination completely abrogates receptor phosphorylation (Y1289) and ATK signaling (S473). For densitometric analysis results are expressed as mean values from three independent experiments. M14 cells were grown in the presence of different doses of vemurafenib **A. right part** or trametinib **B. right part** alone or in combination with 20 μg/ml of anti-ErbB3 mAbs A3, A4 or their combination for 10 days. Cells were then treated as described above. Quantitative analysis, performed by KaleidaGraph software as described above, shows that the combination of A3 and A4 enhances the inhibitory effect of vemurafenib and trametinib on cell growth better than the single mAbs. IC50 vem = 0.35 μM; IC50 vem + A3 = 0.11 μM; IC50 vem + A4 = 0.08 μM; IC50 vem + A3 + A4 = 0, 03 μM. IC50 tram = 0.21 μM; IC50 tram + A3 = 0.08 μM; IC50 tram + A4 = 0.07 μM; IC50 tram + A3 + A4 = 0.02. Results are reported as mean values ± standard deviation (SD) from three independent experiments. *p*-values were calculated using Student's *t* test and significance level has been defined as *p* < 0, 05. For IC50 vem + A3, IC50 vem + A4, IC50 tram + A3 and IC50 tram + A4 *p* < 0, 001 vs IC50 vem and IC50 tram respectively. For IC50 vem + A3 + A4 and IC50 tram + A3 + A4 *p* < 0, 0001 vs IC50 vem and IC50 tram respectively. **C.** Cells were treated with suboptimal doses of vemurafenib and/or trametinib alone or in combination with A3 and/or A4 mAbs. The *in vitro* colony formation assay shows that the addition of mAbs combination inhibits cells growth better than the single mAbs. *p < 0, 01 vs untreated cells; **p < 0, 001 vs untreated cells; NS vs untreated cells.

We next assessed whether the combination of the anti-ErbB3 antibodies A3 and A4 is more potent than single antibody treatment to block vemurafenib-induced activation of the pErbB3/pAKT axis and to synergize with vemurafenib in the inhibition of melanoma cell growth. Total protein extracts of M14 cells exposed to A3, A4 and vemurafenib individually or in combination were subjected to Western Blot analysis. The results (Figure 5A, left panel) clearly showed that while A3 and A4 alone were able to partially inhibit vemurafenib-induced pErbB3 and pAKT activation, only their combination was able to completely abrogate receptor activation and downstream signaling. Notably, we observed that the triple combined treatment leads to a complete abrogation of pERK.

In order to assess whether inhibition of pErbB3 and pAKT could result in potentiation of the growth inhibitory effects of vemurafenib, *in vitro* colony formation assays were carried out in the presence of growing concentrations of vemurafenib alone, in combination with A3 or A4 or with both the mAbs. Treatment with both anti-ErbB3 mAbs vs single mAb treatment strongly potentiated growth inhibition by vemurafenib (12-fold vs 3-fold) at all doses of the BRAF inhibitor. The same findings were confirmed in the WM266 melanoma cell line (Supplementary Figure S5).

Since we have previously demonstrated that the ErbB3-dependent feedback survival loop is activated also by MEK inhibitors [27], we decided to test the anti-ErbB3 mAbs combination with trametinib. To this purpose we performed Western Blot analysis and *in vitro* colony formation assays as described above. As expected also the mAbs combination with trametinib completely abrogated MEK inhibitor-induced ErbB3 and AKT activation (Figure 5B, left panel). Moreover the triple combinations resulted had a stronger impact on melanoma cells growth compared to double treatments (11-fold vs 3-fold). Finally when cells were treated with suboptimal doses of vemurafenib and trametinib, the addition of A3 and A4 mAbs combination was capable to provide a powerful synergistic inhibition of cell growth (Figure 5C).

**Figure 6:**
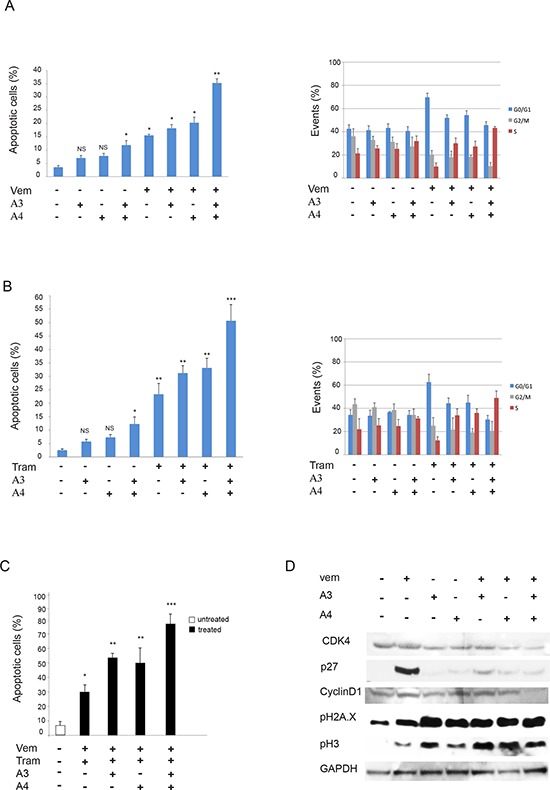
Anti-ErbB3 mAbs combination induces melanoma cell apoptosis better than the single mAbs when combined with vemurafenib and/or trametinib M14 melanoma cells treated with vemurafenib (1 μM) **A. left part** or trametinib (0.5 μM) **B. left part** for 48 h were incubated with 20 μg/ml of anti-ErbB3 mAbs A3, A4 or their combination. Apoptosis induction, evaluated by FACS analysis, shows that the combination of A3 and A4 enhances the apoptotic effect of vemurafenib and trametinib better than the single mAbs. *p < 0, 01 vs untreated cells; **p < 0, 005 vs untreated cells; ***p < 0, 001 vs untreated cells; NS vs untreated cells. M14 cells treated as above for 24 h were assayed for cell cycle analysis by FACS. The results **A and B. right parts** show that combination of A3 and A4 + either vemurafenib or trametinib causes primarily a block in S-phase. **C.** Cells were treated with suboptimal doses of vemurafenib and/or trametinib alone or in combination with A3 and/or A4 mAbs for 48 h. The apoptosis induction, evaluated as above, shows that the addition of mAbs combination enhances the apoptotic effect of the drugs better than the single mAbs. *p < 0, 01 vs untreated cells; **p < 0, 001 vs untreated cells; NS vs untreated cells. *p < 0, 01 vs untreated cells; **p < 0, 005 vs untreated cells; ***p < 0, 001 vs untreated cells. **D.** M14 cells serum starved and treated with vemurafenib (1 μM) for 24 h were incubated with 20 μg/ml of anti-ErbB3 mAbs A3, A4 or their combination. Western blot analysis shows that co-treatment with vemurafenib and A3 + A4 causes a reduction of CDK4 and Cyclin D1 and an increase of the phosphorylation of histone H3 and gamma-H2AX.

In order to provide further insights into the biological effect of mAbs combination with vemurafenib or trametinib we evaluated apoptosis induction by flow cytometry in M14 melanoma cells. Consistently, in cells treated with triple combinations we observed an increased percentage of cells undergoing apoptosis after 48 hours of treatment compared to single and double treatments (Figure 6A and 6B, left panels). Importantly when we treated M14 melanoma cells with the quadruple combination (vemurafenib, trametinib, A3 and A4) we observed a powerful synergistic apoptosis induction (Figure 6C). Moreover, cell cycle analysis showed that, while vemurafenib or trametinib treatments were characterized by a G0/G1 arrest in line with previous literature reports [37, 38], combination of A3 and A4 + either vemurafenib or trametinib caused primarily a block in S-phase (Figure 6A and 6B, right panels), suggestive of the activation of a DNA damage checkpoint. In order to investigate this aspect further, M14 melanoma cells exposed to A3, A4 and vemurafenib alone or their combinations and subjected to Western Blot analysis for a set of cell cycle markers (Figure 6D). Results clearly showed that, while treatment with vemurafenib was accompanied by strong activation of p27 in line with the G0/G1 block, co-treatment with vemurafenib and A3 + A4 caused a dramatic reduction of CDK4 and Cyclin D1, while inducing a strong increase of phosphorylated histone H3 and of gamma-H2AX phosphorylation (pH2AX), thus confirming the occurrence of DNA damage. Of note, similar results were obtained with mAbs combination with trametinib.

### Anti-ErbB3 mAbs combination strongly inhibits “*in vivo*” melanoma cell growth and reduces tumor relapse when combined with vemurafenib and trametinib

We next assessed the effect of the anti-ErbB3 antibodies A3 and A4 on melanoma cell growth *in vivo*.

Firstly we compared the efficacy of single treatment with vemurafenib or with trametinib with that of the combination of A3 + A4. Mice were injected s.c. with 1 × 10^6^ M14 cells. When tumors reached the size of 100 mm^3^ mice were randomly divided into four different groups of 6 mice and subjected to treatments for five weeks. Tumor growth was measured once/weekly. Our data (Supplementary Figure S6A and S6B) showed the capability of the anti ErbB3 antibodies mAbs to partially inhibit melanoma cells growth also *in vivo*.

**Figure 7:**
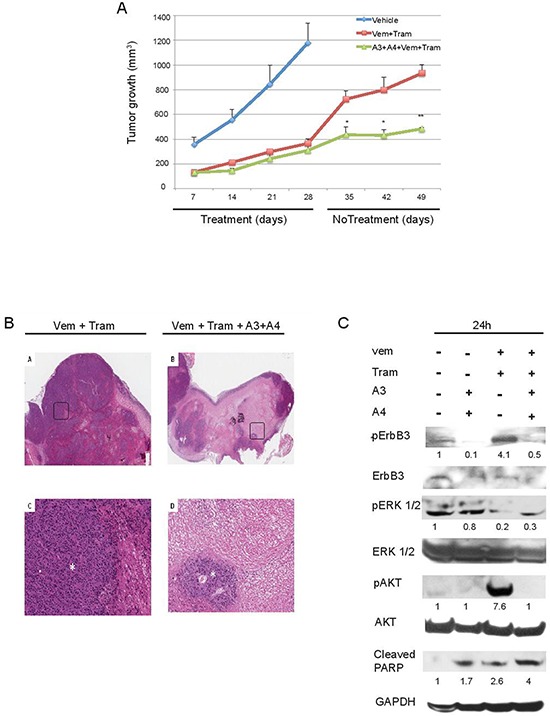
Anti-ErbB3 mAbs combination reduces tumor relapse when combined with vemurafenib and trametinib **A.** M14 melanoma cells were subcutaneously injected in immunodeficient mice at 1 × 10^6^ cells/mouse. Treatments started when tumors reached a 100 mm^3^ volume and mice were allocated six per group. Mice were treated with vehicle, vemurafenib (25 mg/kg) and trametinib (0, 5 mg/kg) alone or in combination with A3 and A4 mAbs (10 + 10 mg/kg) for four weeks. Tumor growth was measured once/weekly. The results show that the quadruple combination group is characterized by a durable control of tumor growth. *p < 0, 01 vs vehicle-treated mice; **p < 0, 005 vs vehicle-treated mice. **B.** Rapresentative images of histology of Vem + Tram-treated mice (left panel) and Vem + Tram + A3 + A4-treated mice (right panel). The quadruple combination-treated ones show a lower percentage of viable areas (asterisk) within the tumor mass and larger sheets of coagulative tumor cell necrosis compared to the Vem + Tram-treated tumors (A-B, H&E, original magnification x 20) (C-D, insert, H&E, original magnification x 200). **C.** Mice with M14 tumors grown subcutaneously were treated with a single dose of the vemurafenib/trametinib combination or with the quadruple combination vemurafenib + trametinib + A3/A4 for 24 h. Mice were then euthanized, tumor collected and total protein extracts analyzed by Western Blot. The results show that vemurafenib + trametinib induce phosphorylation of ErbB3 (Y1289) and AKT (S473) that is abrogated by anti-ErbB3 mAbs also *in vivo*.

Gadiot et al [39] have recently compared the efficacy *in vivo* of single treatment of mutant BRAF melanoma with vemurafenib alone, trametinib alone or the combination of vemurafenib and trametinib. While vemurafenib alone was able to control tumor growth only for a short period of 3–4 weeks after which relapses occur, the combination of vemurafenib and trametinib was able to control cancer growth for a longer time. However, also in these cases, mice were not cured because tumors started to regrow soon after discontinuation of treatment. This mirrors exactly what happens in the clinic. We decided, therefore, to evaluate if combination treatments using anti-ErbB3 mAbs would be able to significantly improve long term survival and better control disease relapse. In order to investigate these aspects we carried out a second *in vivo* study where we compared the efficacy of the combination vemurafenib + trametinib, with that of vemurafenib + trametinib + A3 and A4. The results are shown in Figure 7A panel a. In both groups melanoma cell growth was strongly impaired in a comparable manner, even though the presence of the antibodies showed a modest but not significant trend to superiority. Interestingly, however, when we stopped treatments, while the quadruple combination group showed an evident and durable control of tumor growth, the vemurafenib/trametinib group tumors grew up rapidly. In order to better understand the mechanism at the basis of the differences in the rate of tumor regrowth between these two groups of mice pathology analysis was carried out. The results (Figure 7B and Table 2) show that tumors from mice treated by vemu + tram + A3 + A4 are characterized almost exclusively by large necrotic areas (> 80%) with small residual areas of tumor growth. In contrast in tumors from mice treated by vemu + tram vital areas predominate. Differences between the two groups are highly statistically significant (*p* = 0.004). Furthermore the residual vital areas in the quadruple treatment group show a lower degree of proliferation.

**Table 2:** Tumors treated with vemurafenib and trametinib show a lower tumor necrosis and tumor higher Ki-67 expression compared to tumors treated with the quadruple combination

Group	Necrosis (%)Mean Value	Ki-67 (%)Mean Value
Vem + Tram	61 ± 12.4	79 ± 2, 23
Vem + Tram + A3+A4	82 ± 5.7	71 ± 6, 5

The analysis of tumor necrosis percentage showed a significant difference (*p* = 0.004) between the tumors treated with Vem + Tram (mean value ± standard deviation = 61 ± 12.4; range 40% to 70%) and the tumors treated with Vem + Tram + A3 + A4 (mean value ± standard deviation = 82 ± 5.7; range 75% to 90%). The mean percentage of Ki-67 expression was 79% (range 75% to 80%) in the Vem + Tram-treated tumors and 71% (range 60% to 75%) in the Vem + Tram + A3 + A4 -treated tumors (*p* = 0.016).

Moreover the quadruple treatment with vemurafenib + trametinib + A3 + A4 did not induce higher toxicity over the combination of vemurafenib + trametininb as shown by serial body weight measurements over the course of the study (see Supplementary Figure S7).

Finally to assess the short term effect of *in vivo* treatments we carried out the following pharmacodynamic study. Mice with M14 tumors grown subcutaneously up to the size of 1 cm^3^ were treated with a single dose of the vemurafenib + trametinib combination or with the quadruple combination vemurafenib + trametinib + A3/A4. After 24 hours mice were euthanized, tumor collected total protein extracts analyzed by Western Blot. The results shown in Figure 7C indicate that the strong activation of pErbB3 and pAKT following vemurafenib/trametinib treatments was evident also *in vivo* and was completely abrogated by A3 and A4. Moreover the quadruple combination resulted in a strong inhibition of pERK signaling and in the cleavage of PARP, a marker of apoptosis (Figure 7C). Of note, we subjected the total protein extracts from tumors treated with vehicle or vemurafenib + trametinib to an RTK array to detect early changes in the phosphorylation level of approximately fifty RTKs. According to our previous data [27] we found that, while the phosphorylation level of most receptors, including also PDGFR and IGF1R remained unchanged or underwent minor variations, ErbB3 phosphorylation was prominently increased (Supplementary Figure S8). These data strongly underline the role of ErbB3 in the rebound of melanoma cell growth following vemurafenib/trametinib treatments and pave the way for the use of anti-ErbB3 mAbs as adjuncts to current therapies in order to obtain a durable control of tumor growth.

## DISCUSSION

Therapy of patients with metastatic melanoma has significantly improved over the past years thanks to the introduction of several targeted terapies; however, there is still need for substantial improvements. This is particularly clear in the case of patients bearing mutations in the BRAF oncogene where the initial introduction of BRAF inhibitors, although capable of inducing impressive objective responses in approximately 50% of patients, extended progression free survival (PFS) only up to 5–6 months from the historical 2.5 months previously obtained with dacarbazine [40]. In this set of patients PFS has been further improved to reach approximately 10 months when a BRAF inhibitor is combined with a MEK inhibitor [18–20]. However, disease relapses also in these cases constantly occur because of the emergence of drug resistance, a phenomenon that has been the object of intensive speculations also in the case of other highly selective oncogene kinase inhibitors [41]. Initial analysis of the genetic lansdscape of mutations occurring in patients exposed to both BRAF and MEK inhibitors, has unveiled a highly complex scenario involving multiple genes and factors responsible for the reactivation of the MAPK pathway [42]. It is virtually impossibile to envisage a therapy directed to inhibit all these targets simultaneously. Hence we are far away from the complete control of this aggressive disease and alternative strategies are required to tackle this issue.

The present study is directed to provide an additional option in this direction using a different approach. It has been previously observed that in oncogene addicted melanoma cells some important compensatory survival pathways are being kept under check [9, 34, 42]. These pathways are feedback-reactivated once cells are exposed to inhibitors of the original oncogene, and their reactivation heavily contributes to the failure of the initial therapy. Identification of pivotal compensatory pathways and of their master regulators may offer tremendous opportunities to prevent the development of drug resistance. We believe, based on our data that one of these master regulators in mutant BRAF melanomas, is indeed the ErbB3 receptor. This conclusion stems from previous observations by our group showing in RTK array studies in three different cell lines that ErbB3 is the main RTK undergoing heavy phosphorylation in response to exposure to a BRAF or a MEK inhibitor [27], and is strengthened in this study in which we show in 7 out of the 8 additional BRAF mutated cell lines that the same degree of ErbB3 activation takes place. Most importantly, also in an *in vivo* mouse xenograft model with one of the new cell lines under study, we observe a prominent ErbB3 hyperphosphorylation as compared to all other receptors analyzed, which confirms the previous *in vitro* findings. Interestingly, however, in the *in vivo* model additional RTKs undergo phosphorylation as compared to *in vitro* which we believe is the result of the contribution of the tumor microenvironment. It has to be stressed that in our experiments we do never observe increased phosphorylation of other RTKs previously reported to be involved in the activation of survival pathways in melanoma cells, such as PDGFR and IGF1R [11, 14]. We don't have an easy explanation for this discrepancy; a major difference in our experimental model is that we analyze RTK changes taking place in the first 24–48 hours after drug exposure, and not after resistance has occurred weeks after cells have been exposed to increasing drug concentrations. Hence it is possible that indeed ErbB3 is the principal receptor involved in early adaptive responses of melanoma cells upon exposure to inhibitors of the MAPK pathway. This evidence if further strengthened by two other observations. The first being the difficulty to generate cells double resistant to a BRAF inhibitor and an anti-ErbB3 antibody, at least at the concentrations used in this study. The second is the evidence that in cells resistant to vemurafenib, the same ErbB3-induced survival loop is still present but is activated in this case by a MEK inhibitor. Most importantly, its inhibition with an anti-ErbB3 antibody rescues completely sensitivity to kinase inhibitors.

What is the mechanism responsible for ErbB3 hyperphosphoryation? It has been previously proposed that BRAF mutated cancer cells either of melanoma or of thyroid or of colorectal origin, undergo increased ErbB3 protein expression consequent to transcripional activation [25, 26]. Various transcriptional regulators have been postulated to be responsible for this effect such as increased binding of the positive factor FOXD3 in melanoma [26] or decrease binding of the negative factor CTBP1 in thyroid cancer [25]. In contrast to previous observations our data show in 10/11 melanoma cell lines that ErbB3 protein levels do not increase (both *in vitro* and *in vivo*) but that there is instead a selective enhancement of its phosphorylation. Extensive RT-PCR analysis does not show any detectable increase in ErbB3 mRNA levels in any of the cells analyzed in contrast with a constant increase in the mRNA levels of its ligand neuregulin. This finding strengthens our previous observations that pointed to the activation of an autocrine survival loop, and therefore to a modified tumor microenvironment. In the future it will be important to unravel the mechanisms at the basis of the increased transcriptional activation of the neuregulin gene, because they may offer additional opportunities for therapeutic intervention.

The ErbB3 receptor is a potent activator of donwstream signaling due to the presence of seven thyrosines in its intracytoplasmic domain, which can serve as docking sites for PI3K when phosphorylated [43]. ErbB3 activation can occur in a variety of ways, which can be distinguished in canonical, namely ligand-dependent heterodimerization with other receptors of the same ErbB family, in particular ErbB2 [44] and non canonical, i.e. through higher order and often ligand independent receptor clustering with other RTKs, such a cMet, and IGF1R [45, 46]. This complex and heterogeneous pattern of activation makes the ErbB3 receptor particularly resilient to inhibition and challenging from the therapeutic standpoint. Since the receptor lacks potent intrinsic kinase activity, the most viable approach to inhibit its function is through the use of monoclonal antibodies [30]. However, also in this case a single monoclonal antibody may not be able to fully abrogate receptor activity and to exert the most potent therapeutic effect. In this paper we use two anti-ErbB3 mAbs directed against two different surface epitopes, one capable of inhibiting ligand-dependent receptor activation, A3 [28], the other, A4, capable of inhibiting both ligand-dependent and ligand-independent receptor activation [29, 30]. The two antibodies when combined together *in vitro* show increased ability to internalize the receptor and to induce its degradation. We believe the extent and duration of receptor degradation is the most useful pharmacodynamic biomarker of inhibition. Our results confirm for ErbB3 what has been previously shown also for EGFR and ErbB2, i.e. that in order to fully inhibit the activity of a receptor of the ErbB family a single antibody is not enough and that a superior efficacy is achieved both *in vitro* and *in vivo* by combining two antibodies against distinct epitopes [36, 47, 48]. Although clinical translation of this concept is highly challenging due to increasing costs of production and to the complexity of clinical development, one has to be aware that a single antibody for a given target will never be able to achieve maximal therapeutic efficacy.

We have been the first to show that activation of the ErbB3/AKT axis in melanoma can be inhibited by co-treatment with anti-ErbB3 mAbs and that these antibodies synergize with BRAF and MEK inhibitors in short term *in vitro* clonogenic assays [27]. This was also confirmed by others [49]. In this paper we provide strong evidence both *in vitro* and *in vivo* that addition of anti-ErbB3 antibodies to the BRAF/MEK combination, suppresses the development of resistance *in vitro* and potently mitigates disease recurrence *in vivo*. This is in line with the finding that A3 and A4 also induce further inhibition of pERK, even when combined with vemurafenib + trametinib. This finding has important clinical implications because Bollag et al [50] have demonstrated that profound inhibition of MAPK transcriptional output correlates to maximal effectiveness of therapy in melanoma patients. Interestingly, *in vitro* studies show that the mechanism of action of anti-ErbB3 antibodies differs from that of MEK and BRAF inhibitors and dominates in the triple combination regimen. In fact, while BRAF and MEK inhibitors block cell cycle progression at the G0/G1 level, combination with anti-ErbB3 mAbs arrests cells in the S-phase, which suggest the activation of a DNA damage checkpoint, further confirmed *in vivo* by strong PARP cleavage. This interesting observation may open up to additional synergies with agents capable to inhibit DNA repair and which so far have not been taken into consideration in melanoma.

Several anti-ErbB3 monoclonal antibodies are under development and have been brought to clinical trials for a variety of indications such as breast, colorectal and ovarian cancers, given the known role of ErbB3 in resistance to therapy [30, 51, 52]. In this context, our data warrant the use of these antibodies in trials of triple combination with a BRAF and a MEK inhibitor with the intent to further improve response rates and achieve more durable disease control.

## MATERIALS AND METHODS

### Cell lines and treatments

Human melanoma cell lines MST-L, WM266, LOX IMVI, M14, A375, MALME 3-M, M229, M262, M263, M397, WM115 were cultured in RPMI supplemented with 10% FBS. To evaluate ErbB3, AKT and ERK 1/2 signaling and neuregulin (HRG) expression melanoma cells were serum starved for 24 h and treated with vemurafenib and/or trametinib at different doses and time-points and incubated or not with 20 μg/ml of anti-ErbB3 mAbs A4, A3 alone or in combination. To determine the effects on melanoma proliferation, seeded at 1 * 10^5^/well, cell lines were treated with increasing concentrations (from 0, 002 to 1 μM) of vemurafenib and/or trametinib alone or in combination with anti-ErbB3 mAbs for 10 days. To generate vemurafenib-resistant WM266 human melanoma cells, they were treated for about two months with increasing drug concentrations every two weeks (from 50 nM to 10 μM). To analyze the possible internalization and degradation of ErbB3, cells were stimulated with HRG at 100 ng/ml or incubated with A3, A4 alone or in combination at 37°C for different time as indicated. For the visualization of the cell surface, plasma membranes were labeled with Vybrant DiL cell labeling solution for 1 h at 4°C before fixation. To induce LysoTracker internalization, cells were incubated with LysoTracker-Red for 1 h at 37°C and then fixed. For proliferation assays, cells were incubated with the anti-ErbB3 monoclonals for 1 h at 37°C and then stimulated with 30 ng/ml HRG for 48 or 72 h.

### Antibodies and reagents

Antibodies against AKT, ERK 1/2, phospho-ErbB3 (Y1289), phospho-AKT (S473), phospho-ERK 1/2 (T202/204), anti-pH2A.X, anti-pH3 and anti-cleaved-PARP were purchased from Cell Signaling Technology. Anti-ErbB3, anti-P27, anti-Cyclin D1, anti-CDK4, anti-UBI and anti-GAPDH were obtained from Santa Cruz Biotechnology. Anti-rabbit and anti-mouse were purchased from AbCam. Anti ErbB3 antibodies A3 and A4 have been described previously [28–30]. The two anti-ErbB3 antibodies are all of the IgG1 isotype (EM unpublished observation). Vemurafenib and trametinib were obtained from Selleck Chemicals. TaqMan probes for ErbB3, NRG1 and housekeeping gene 18S were purchased from Applied Biosystems. The rabbit anti-Ki67 polyclonal antibodies were from Zymed Laboratories. FITC-conjugated goat anti-mouse IgG was obtained from Cappel Research Products. Texas Red-conjugated goat anti-rabbit IgG were from Jackson Immunoresearch Laboratories. DAPI was purchased from Sigma. Vybrant DiI cell labeling solution was from Invitrogen. LysoTracker-Red was obtained from Molecular Probes.

### Immunofluorescence analysis

Melanoma cells, grown on coverslips and incubated with the monoclonals A3, A4 alone or in combination and/or stimulated with HRG were fixed with 4% paraformaldehyde in PBS for 30 min at 25°C followed by treatment with 0.1 M glycine for 20 min at 25°C and with 0.1% Triton X-100 for an additional 5 min at 25°C to allow permeabilization. To evaluate cell proliferation, cells were then incubated for 1 h at 25°C with the rabbit anti-Ki67 polyclonal antibodies. The primary antibodies were visualized using goat anti-mouse IgG-FITC and goat anti-rabbit IgG-Texas Red for 30 min at 25°C. To induce LysoTracker internalization, cells were incubated with LysoTracker-Red for 1 h at 37°C and then fixed. Nuclei were stained with DAPI. Coverslips were finally mounted with mowiol for observation. To assess the extent of colocalization of fluorescence signals, cells were scanned in a series of 0.5 μm sequential sections with an ApoTome System (Zeiss) connected with an Axiovert 200 inverted microscope (Zeiss); image analysis was then performed by the Axiovision software and 3D reconstruction of a selection of three central out of the total number of the serial optical sections was shown in each figure. Quantitative analysis of the extent of colocalization was performed using Zeiss KS300 3.0 Image Processing system. The mean ± standard error (SE) percent of colocalization was calculated, analyzing a minimum of 50 cells for each treatment randomly taken from three independent experiments. Percentage of Ki67-positive cells was obtained counting for each treatment a total of 500 cells, randomly observed in 10 microscopic fields from three different experiments. Results have been expressed as mean values ± standard errors (SE). *p*-values were calculated using Student *t*-test, and significance level has been defined as *p* < 0.05.

### Western blot analysis

Melanoma cells were lysed with RIPA buffer; 50 μg of total protein were resolved under reducing conditions by 8% SDS-PAGE and transferred to reinforced nitrocellulose (BA-S 83, Schleider and Schuell, Keene, NH, USA). The membranes were blocked with 5% non fat dry milk in PBS 0.1% Tween 20, and incubated with the different primary antibodies. The membranes were rehydrated and probed again with anti-GAPDH, to estimate the protein equal loading. Densitometric analysis was performed using Quantity One Program (Bio-Rad Laboratories GmbH) and results were expressed as mean values from three independent experiments.

### Phospho-RTK array

A human phospho-RTK array (R&D Systems) was used to detect simultaneously the phosphorylation status of RTKs (*n* = 49) in melanoma cells. Membranes were incubated with cell lysates (100 μg) overnight according to the manufacturer's protocol. After washing, the membranes were incubated with a phosphotyrosine antibody conjugated to horseradish peroxidase to allow the detection of captured RTKs that are phosphorylated. Duplicate dots in each corner are positive controls.

### RNA extraction and real-time PCR analysis

RNA was extracted using TRIzol method (Invitrogen) according to manufacturer's instruction and eluted with 0, 1% diethylpyrocarbonate (DEPC)- treated water. Total RNA was quantitated by spectrophotometry. Real Time-PCR was assayed by TaqManW Gene Expression Assays (Applied Biosystems, Foster City, CA). To normalize the amount of source RNA, 18S transcript from the same sample was measured and used as internal reference. Each targeted transcript was validated using the comparative Ct method for relative quantification (ΔΔCt) reference to the amount of a common reference gene (18S). The fold difference was calculated using the comparative ΔΔCt and results were reported as mean values from three independent experiments.

### *In vitro* colony formation assay

Cells viability was determined by crystal violet staining. Briefly, the cells were stained for 20 min at room temperature with staining solution (0, 5% crystal violet in 30% methanol), washed four times with water and then dried. Cells were then dissolved in a Methanol/SDS solution and the adsorbance (595 nm) was read using a microplate ELISA reader.

### Statistical analysis

Quantitative analyses for curve fitting and for IC50 evaluation were performed by KaleidaGraph and by GraphPad softwares. The evaluation of Combination Index (CI) was performed by CalcuSyn software using the median effect methods described by T-C Chou and P. Talalay [53]. Briefly, two molecules are synergic if the CI < 1, additive if CI = 1 and antagonist if CI > 1. *p*-values were calculated using Student's *t* test and significance level has been defined as *p* < 0.05.

### Annexin V binding assay

Annexin V binding assay was performed to quantify apoptosis in cells treated with vemurafenib, trametinib, A3 and A4 alone or in combination. Briefly, 1 × 10^6^ cells were treated for 24 hrs with A3 and/or A4 at 20 μg/ml and drugs at variable concentration. After the incubation period, cells were washed in PBS and stained with 5 μl of Annexin V-FITC (Invitrogen) and PI at 5 μg/ml (Invitrogen) for 15 minutes at room temperature in the dark. Apoptosis were determined using MACSquant cytofluorometer. Both early (Annexin V-positive, PI negative) and late (Annexin V-positive, PI positive) apoptotic cells were included in cell death determination.

### Cell cycle analysis

Cell cycle analysis was performed in cells treated or not with A3, A4 alone or in combination at 20 μg/ml and/or vemurafenib/trametinib at variable concentration. The cell cycle kit, purchased from Millipore, was used according to the manufactures' instructions.

### *In vivo* efficacy studies

All studies have been performed in accordance with the “Directive 2010/63/UE” on the protection of animals used for scientific purposes, made effective in Italy by the Legislative Decree 4 March 2014, n.26, and applying the principles of 3Rs, i.e. replace, reduce, refine. Mice were purchased at Harlan Laboratories (Udine, Italy). After 1 week of acclimatization they were housed five to a plastic cage and fed on basal diet (4RF24, Mucedola S.r.l.) with water *ad libitum*, in an animal facility controlled at a temperature of 23 ± 2°C, 60 ± 5% humidity, and with a 12 h light and dark cycle. All procedures performed on the animals were approved by Animal Welfare Body and authorized by the Italian Ministery of Health, 46/2014-PR. At the end of the treatment period and before necropsy, mice were euthanized by compressed CO_2_ gas in cylinder as indicated in the AVMA (American Veterinary Medical Association) Panel on Euthanasia and according to the guidelines described in (UKCCR, 1998.). M14 melanoma cells were subcutaneously injected in immunodeficient mice at 1 × 10^6^ cells/mouse. Cell were resuspended in a 50% RGF matrigel (BD Biosciences) solution in PBS and injected in the right flank of the mice in 100 μl volume. Treatments started when tumors reached a 100 mm^3^ volume and mice were allocated six per group. Mice were treated with vehicle (1%Tween80/1%beta-HPCD), vemurafenib at 25 mg/kg, trametinib at 0, 5 mg/kg, A3 and A4 in PBS at 20 mg/kg, or combination of the four. Vemurafenib and trametinib were dosed p.o., daily, 5 days/week, while A3 and A4 antibodies were dosed i.p., once per week and treatment lasted for 4 weeks. At the end of the treatment mice were euthanized and, after harvesting, tumor weight was determined and tumor masses used for further analysis.

### Histology and immunohistochemistry

Tumor samples from mice were frozen and then formalin fixed and paraffin embedded (FFPE). Haematoxylin and eosin slides were used to evaluate the percentage of tumor necrosis. Immunohistochemistry for Ki-67 (clone MIB-1, Dako, Denmark) was carried out using the Dako automated immunostainer (DAKO, Denmark). Tumor proliferation index was assessed as the percentage of Ki-67-positive tumor nuclei.

## SUPPLEMENTARY FIGURES and TABLE


