# Supplementary material for: Combination of antibodies directed against different ErbB3 surface epitopes prevents the establishment of resistance to BRAF/MEK inhibitors in melanoma
Source: Oncotarget. 2015 Jul 7;6(28):24823–41. doi: 10.18632/oncotarget.4485 (PMC4694796; doi:10.18632/oncotarget.4485)
Supplement: Supplementary file 1 [file oncotarget-06-24823-s001.pdf]

SUPPLEMENTARY FIGURES AND TABLE

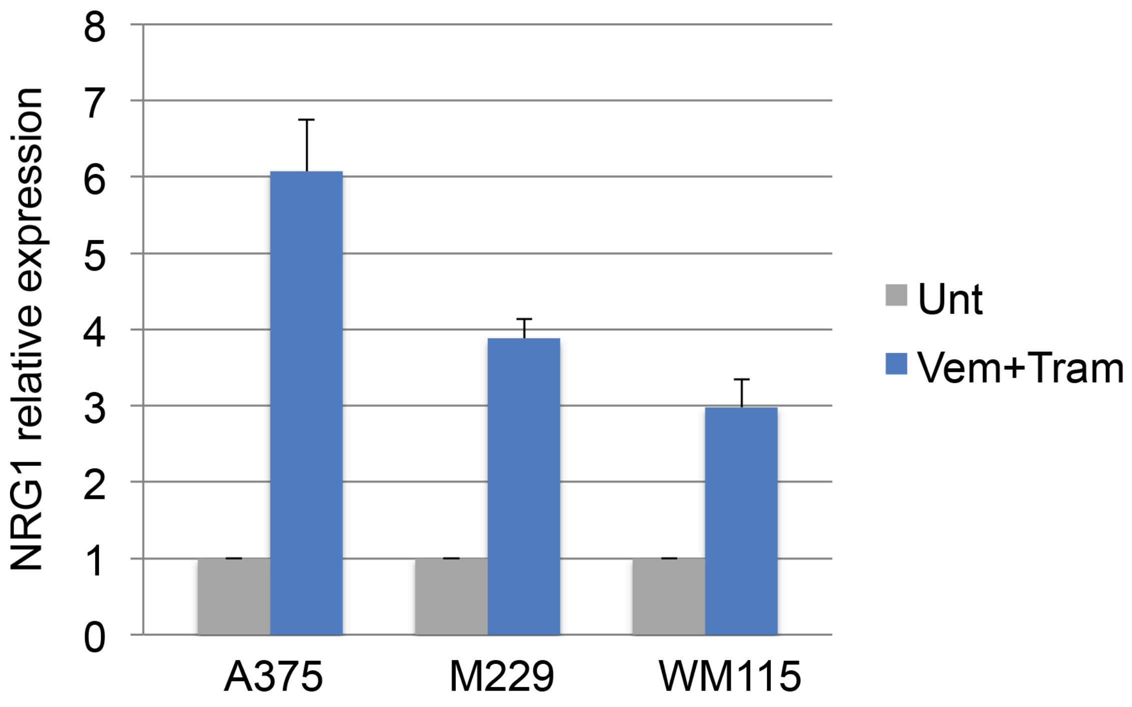

Supplementary Figure S1: Combination treatments of vemurafenib and trametinib increase NRG1 expression in melanoma cells. Melanoma cells were serum starved for 24 h and then treated or not with vemurafenib (0, 3 µM) and trametinib (0.15 µM) for 24h. Real-time PCR analysis, performed using specific TaqMan probes, show that treatments induce an increase of mRNA relative to NRG1. For PCR analysis results are reported as mean values +/− standard deviation (SD) from three independent experiments.

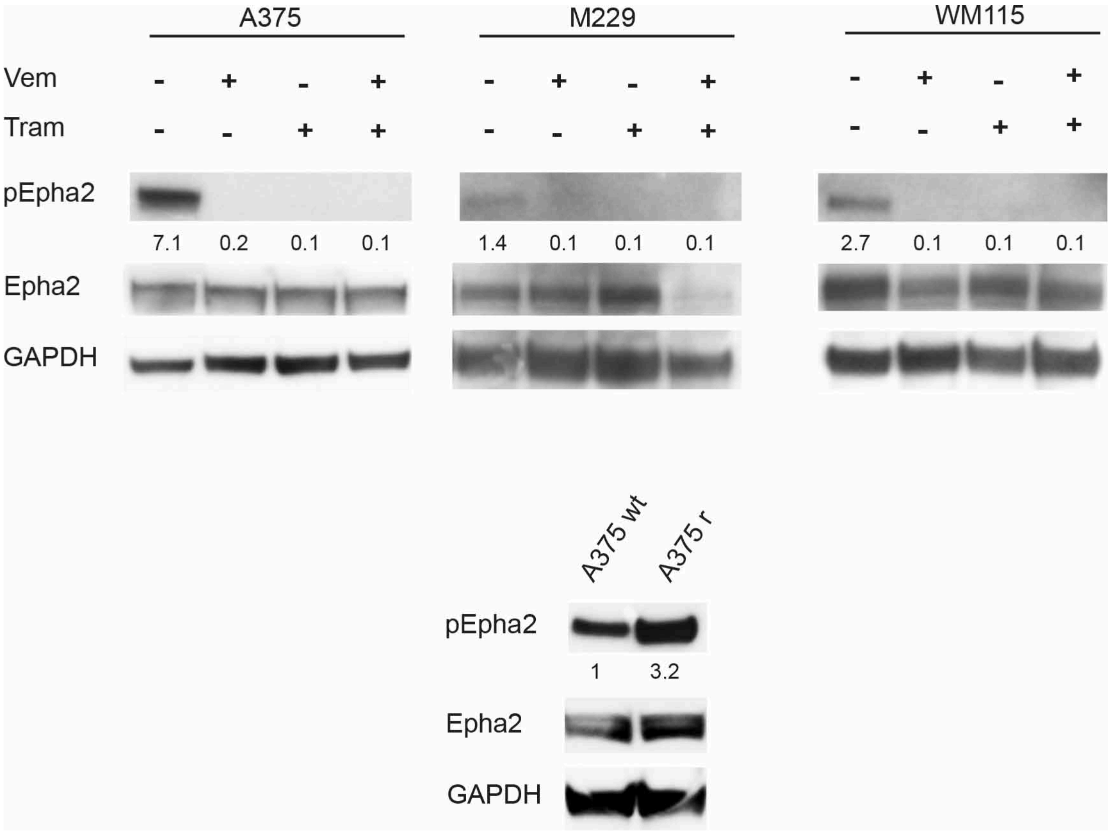

Supplementary Figure S2: Vemurafenib and/or Trametinib treatments reduce Epha2 phosporylation in melanoma cells. A375, M229 and WM115 cells were serum starved for 24 h, treated or not with vemurafenib (0.3 μM), with trametinib (0.15 μM) or with their combination for 24 h. Western blot analysis performed using the indicated antibodies shows that both vemurafenib and trametinib reduce Epha2 phosphorylation (S897). In contrast vemurafenib-resistant A375 cells (A375 r) show strong Epha2 receptor activation compared to wild type ones (A375 wt). For densitometric analysis results are expressed as mean values from three independent experiments.

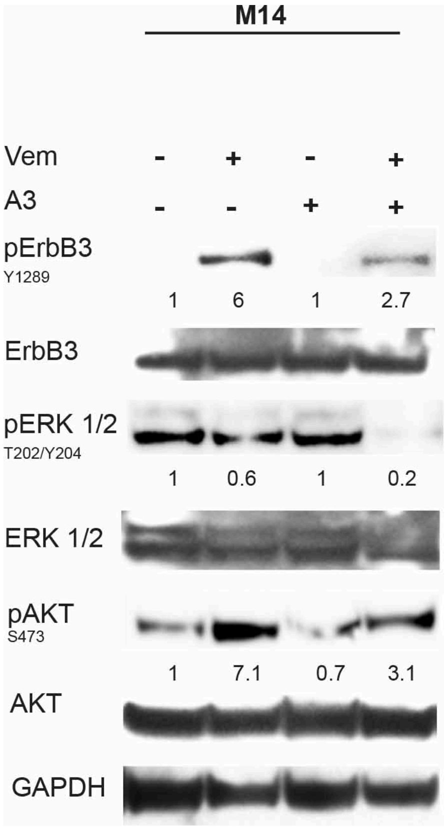

Supplementary Figure S3: Anti-ErbB3 A3 mAb counteracts the increase of ErbB3-dependent AKT phosphorylation. M14 cells serum starved and treated with vemurafenib (0.3 μM) for 24 h were incubated or not with A3 mAb (20 μg/ml). Western blot analysis shows that A3 abrogate receptor phosphorylation (Y1289) and ATK (S473) signaling. For densitometric analysis results are expressed as mean values from three independent experiments.

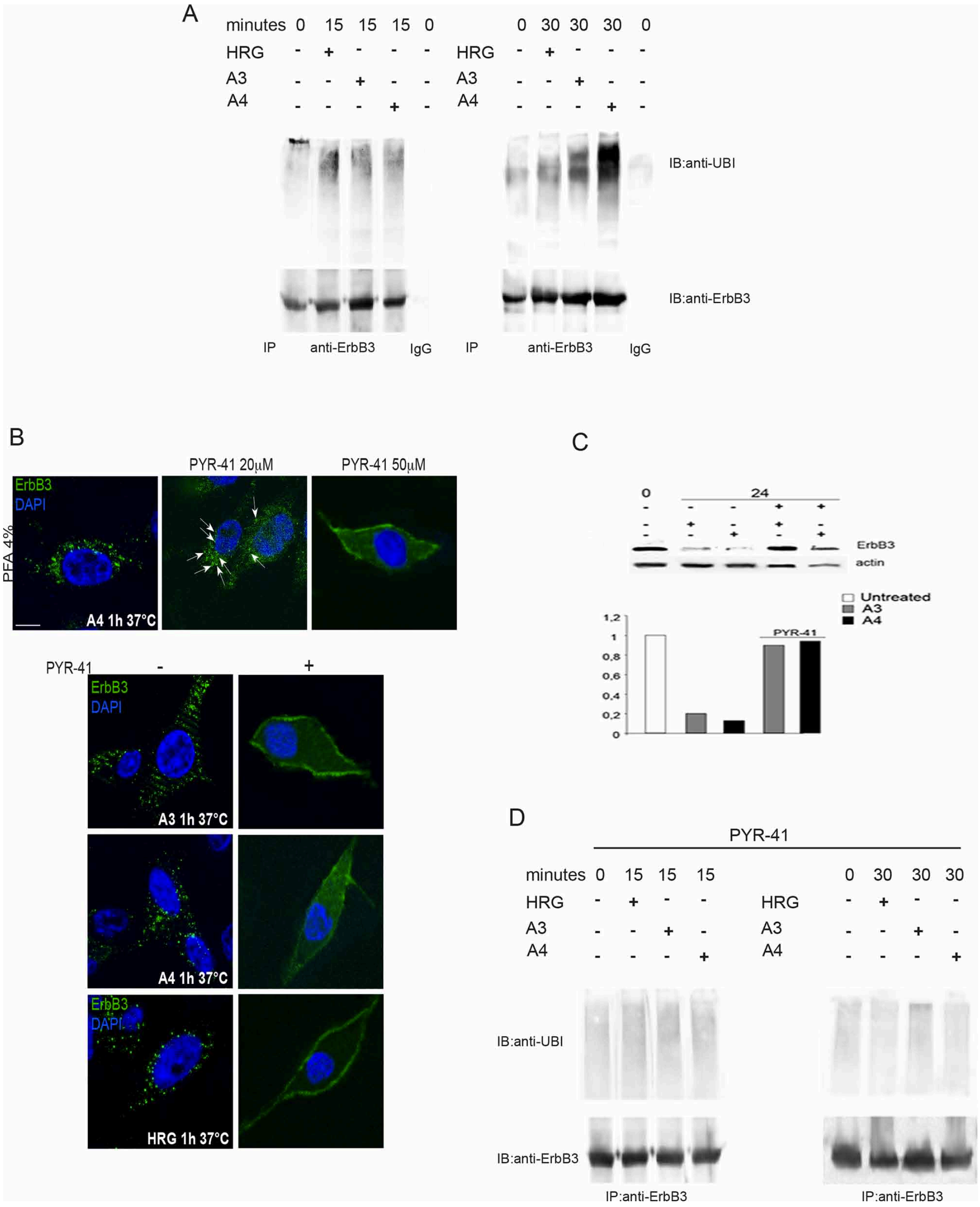

Supplementary Figure S4: Anti-ErbB3 mAbs induce ubiquitination-dependet receptor internalization and degradation. **A.** MST-L cells were treated with NRG1 (HRG), A3 or A4 for different time points and the total protein extracts subjected to immunoprecipitations with an antibody directed against the ErbB3 receptor. Western Blot analysis using anti-UBI antibodies show that anti-ErbB3 mAbs induce strong receptor ubiquitination after 30 minutes of treatment compared to HRG stimulation. **B.** MST-L cells were exposed to the ubiquitination-inhibitor PYR-41 for 1 h and then treated with A3, A4 or NRG1 for 1 h at 37°C before fixation to allow endocytosis of antibody-receptor complexes. Immunofluorescence analysis was performed using a secondary anti-mouse FITC antibody. The signal of A3, A4 or HRG appears clustered in endocytic dots. Differently, PYR-41 (at 50 μM) blocks mAbs and ligand-induced receptor internalization because the signal remains uniformly distributed on the plasma membrane. Bar = 10 μm. **C.** Western blot analysis using anti-ErbB3 antibodies were performed in MST-L cells exposed or not to PYR-41 for 1 h and then treated with A3 or A4 for 24 h. The results show that PYR-41 pre-treatments are able to inhibit mAbs-induced ErbB3 degradation. The equal loading was assessed as described before. **D.** MST-L cells exposed or not to PYR-41 for 1 h were treated with NRG1, A3 or A4 for different time points and the total protein extracts subjected to immunoprecipitations as described above. Western Blot analysis show that PYR-41 pre-treatments inhibit mAbs-induced receptor ubiquitination.

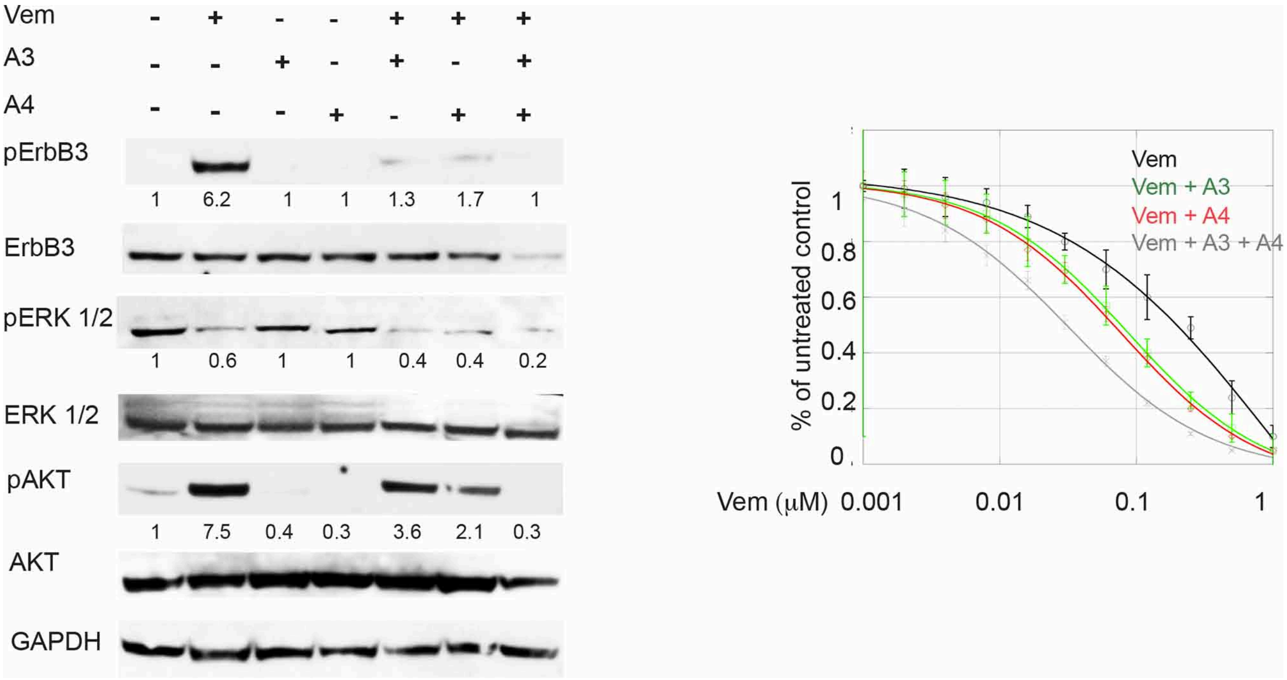

Supplementary Figure S5: Anti-ErbB3 mAbs combination counteracts the increase of receptor phosphorylation and potentiate growth inhibition induced by vemurafenib better than the single mAbs. WM266 cells serum starved and treated with vemurafenib (0.3 μM) (**left part**) for 24 h were incubated with 20 μg/ml of anti-ErbB3 mAbs A3, A4 or their combination. Western blot analysis shows that only mAbs combination completely abrogates receptor phosphorylation (Y1289) and ATK signaling (S473). For densitometric analysis results are expressed as mean values from three independent experiments. WM266 cells were grown in the presence of different doses of vemurafenib (**right part**) in combination with 20 μg/ml of anti-ErbB3 mAbs A3, A4 or their combination for 10 days. Cells were then treated as described above. Quantitative analysis, performed as described above, shows that the combination of A3 and A4 enhances the inhibitory effect of vemurafenib on cell growth better than the single mAbs. IC50 vem = 0.16 μM, IC50 vem + A3 = 0.075 μM; IC50 vem + A4 = 0.065 μM; IC50 vem + A3 + A4 = 0.023 μM. Results and *p*-values are calculated as described above. For IC50 vem + A3 and IC50 vem + A4 *p* < 0, 001 vs IC50 vem. For IC50 vem + A3 + A4 *p* < 0, 0001 vs IC50 vem.

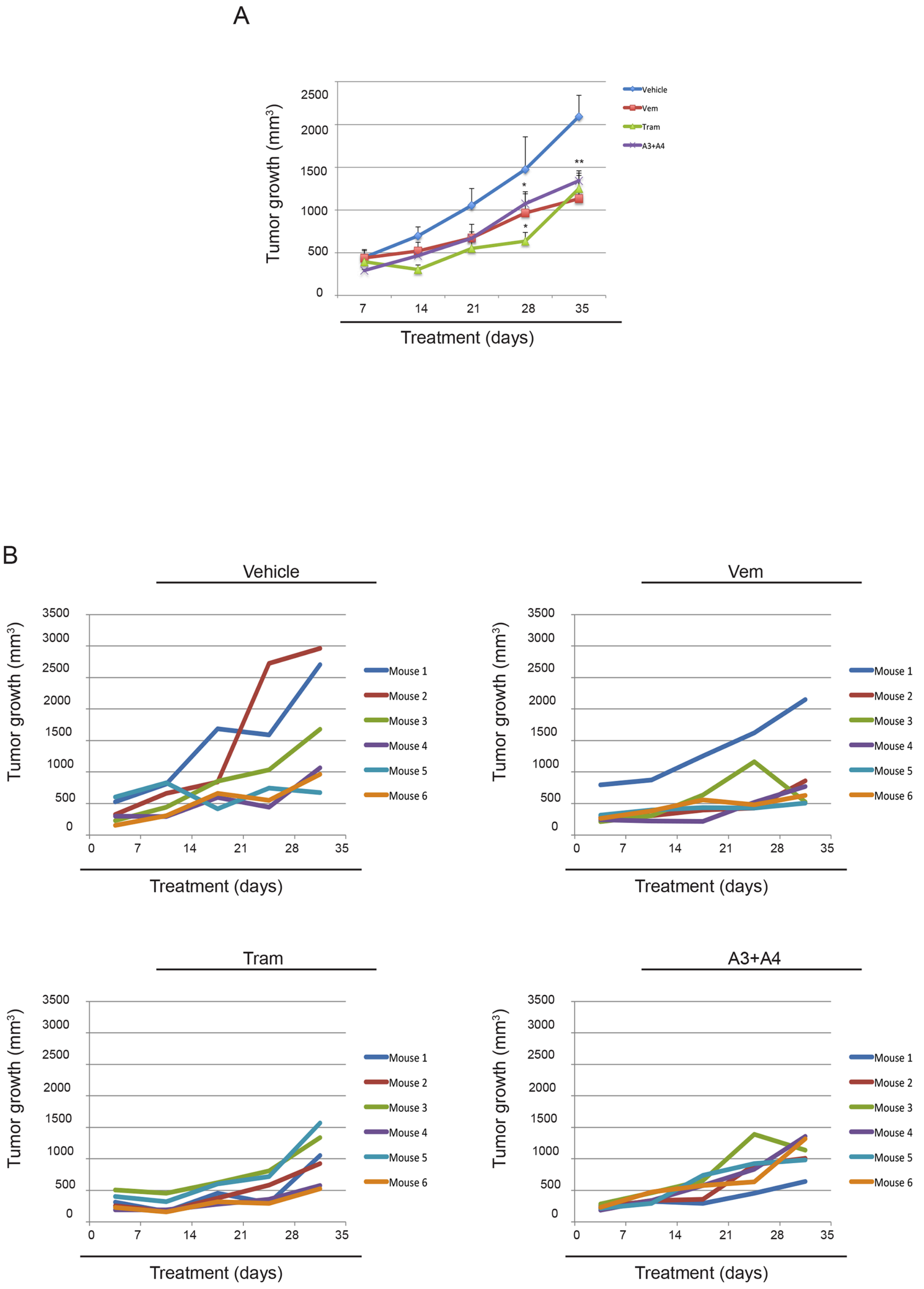

Supplementary Figure S6: Anti-ErbB3 mAbs combination inhibits "*in vivo*" melanoma cell growth. A. M14 melanoma cells were subcutaneously injected in immunodeficient mice at 1×10^6 cells/mouse. Treatments started when tumors reached a 100 mm^3 volume and mice were allocated six per group. Mice were treated with vehicle, vemurafenib (25 mg/kg), trametinib (0, 5 mg/kg) or A3/A4 mAbs combination (10+10 mg/kg) for five weeks. Tumor growth was measured once/weekly. The results show the capability of the anti ErbB3 antibodies mAbs to partially inhibit melanoma cells growth also *in vivo*. *$p < 0, 01$ vs vehicle-treated mice; **$p < 0, 005$ vs vehicle-treated mice. **B.** The measurements performed in the single mice are reported.

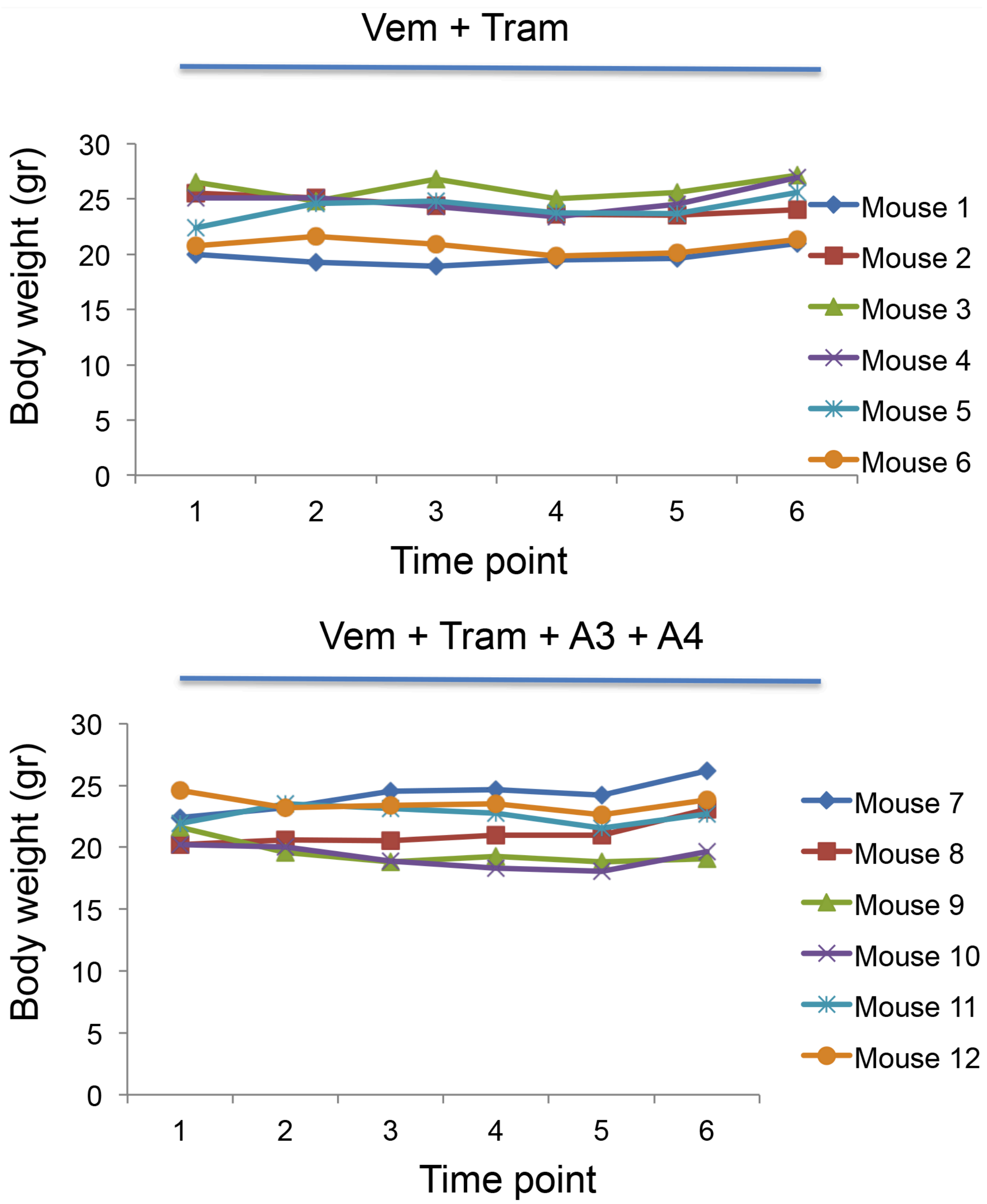

Supplementary Figure S7: Anti-ErbB3 mAbs combination with vemurafenib and trametinib did not alter mice body weight compared to vemurafenib/ trametinib treated mice. M14 melanoma cells were subcutaneously injected in immunodeficient mice and treated as described above. Mice body weight was monitored at different time points. The results show that the quadruple combination treated group has not difference in mice body weight compared to vemutafenib + trametinib treated group.

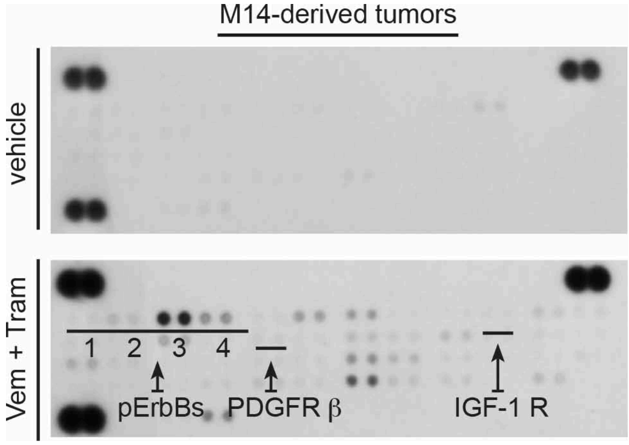

Supplementary Figure S8: ErbB3 is the most activated receptor upon exposure to vemurafenib/trametinib treatments also "*in vivo*". Simultaneous detection of the phosphorylation status of RTKs (n = 49) using a human phospho-RTK array in M14 cells derived protein extracts obtained as described in Figure 7 (part b). Membranes were incubated with cell lysates (100 µg) overnight according to the manufacturer's protocol. The array detects the tyrosine-phosphorylated RTKs simultaneously in duplicate (1, pErbB1; 2, pErbB2; 3, pErbB3; 4, pErbB4). Duplicate dots in each corner are positive controls. Array data on images were analyzed using Photoshop Quantity One Program (Bio-Rad LaboratoriesGmbH). The phosphorylation of ErbB3 is increased by vemurafenib/trametinib treatments also "*in vivo*".

Supplementary Table S1: Vemurafenib treatment increases NRG1 expression in melanoma cells.

Melanoma cells were serum starved for 24 h and then treated or not with vemurafenib (0, 3 µM) for 24 h. Real-time PCR analysis, performed using specific TaqMan probes, show that while vemurafenib treatment induces an increase of mRNA relative to NRG1, ErbB3 receptor expression is unchanged or slightly decreased. For PCR analysis results are reported as mean values ± standard deviation (SD) from three independent experiments.

Cell line	ErbB3	NRG1
WM115	0, 63 ± 0, 12	7, 03 ± 0, 16
M229	1, 08 ± 0, 19	2, 26 ± 0, 48
WM266	1, 3 ± 0, 11	3, 39 ± 1, 08
A375	1, 4 ± 0, 38	5, 51 ± 0, 74
LOX IMVI	1, 25 ± 0, 47	2, 3 ± 0, 28
M14	0, 55 ± 0, 03	1, 71 ± 0, 25
